# Tissue Distribution and Biochemical Changes in Response to Copper Accumulation in *Erica australis* L.

**DOI:** 10.3390/plants10071428

**Published:** 2021-07-13

**Authors:** Daniel Trigueros, Sabina Rossini-Oliva

**Affiliations:** Department of Plant Biology and Ecology, University of Seville, Avda. Reina Mercedes s/n, P.O. Box 1095, 41012 Seville, Spain; biodtv@hotmail.com

**Keywords:** tolerance, Ericaceae, mining, toxicity

## Abstract

Copper uptake, accumulation in different tissues and organs and biochemical and physiological parameters were studied in *Erica australis* treated with different Cu concentrations (1, 50, 100 and 200 µM) under hydroponic culture. Copper treatments led to a significant reduction in growth rate, biomass production and water content in shoots, while photosynthetic pigments did not change. Copper treatments led to an increase in catalase and peroxidase activities. Copper accumulation followed the pattern roots > stems ≥ leaves, being roots the prevalent Cu sink. Analysis by scanning electron microscopy coupled with elemental X-ray analysis (SEM–EDX) showed a uniform Cu distribution in root tissues. On the contrary, in leaf tissues, Cu showed preferential storage in abaxial trichomes, suggesting a mechanism of compartmentation to restrict accumulation in mesophyll cells. The results show that the studied species act as a Cu-excluder, and Cu toxicity was avoided to a certain extent by root immobilization, leaf tissue compartmentation and induction of antioxidant enzymes to prevent cell damage.

## 1. Introduction

A heavy metal such as Cu is an essential nutrient being required for normal plant growth for several biochemical processes as a constituent of enzymes and proteins. However, a high Cu concentration in the soil resulting in toxicity levels may occur when parental materials have been Cu-rich, and soil pH favors metal availability, or polluted by mining activities and waste deposits, or in agricultural soils by intensive use of Cu-containing compounds for plant disease control or heavy application of manure or sewage sludge [1,2]. The critical toxicity level for most crops is above 20–30 mg kg^−1^ leaf dry weight [3], while in Cu-tolerant metallophytes, leaves may contain up to 1000 µg g^−1^ leaf dry weight [4,5]. Leaf chlorosis and stunted growth are the more frequent copper toxicity symptoms observed mostly as the result of inhibition of nutrient uptake or direct interference with plant metabolism [2,6].

A significant body of knowledge about heavy metal tolerance in plants has been acquired from the study of species thriving in the harsh environments of abandoned mines [3,7,8]. In the Iberian Pyrite Belt, a sulphide mining area of southern Portugal and SW Spain, *Erica australis* shares habitat with *E. andevalensis* while growing on highly acidic and heavy-metals polluted soils [9,10,11]. In these soils, Cu appears at a concentration of up to 1400 mg kg^−1^ [11]. However, plant species growing in such contaminated soils hardly reach toxic Cu concentration in their tissues [9,11,12].

The (first barrier) main strategy to tolerate heavy metal stress is to reduce metal uptake and transport by root fixation or complexation at the rhizosphere [3,7]. If high Cu levels reach the leaves, the metal has to be complexed and stored in vacuoles to avoid the production of reactive oxygen species (ROS). Meanwhile, increased activity of ROS scavenging systems is induced to cope with free radicals, which might generate protein damage by oxidative stress [13]. Organic compounds such as amino acids, amides and carboxylates, of known metal-complexing properties are synthesized in response to metallic stresses [6]. However, their roles in metal tolerance still need to be proved in many species.

Some of these mechanisms related to the Cu tolerance, such as metal fixation in roots, induction of antioxidant systems and increased in organic complexing compounds, have been described for *E. andevalensis* [14]. However, *E. australis* colonizes much wider areas in the same polluted soils of Riotinto. Might it be because of differential sensitivity to metal excess or greater nutrient use efficiency? The aim of the present work was to study how an excess of Cu in *E. australis* might affect plant growth and biochemical parameters in comparison with other species that successfully thrive on heavy metal polluted soils.

## 2. Results

**Figure 1:**
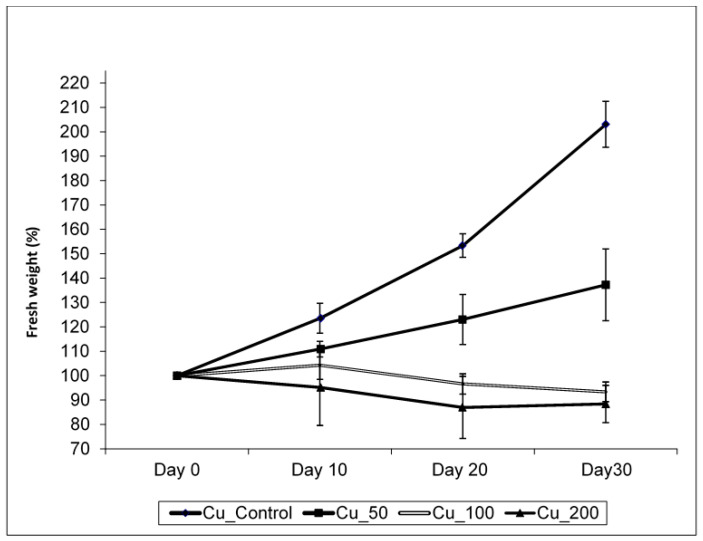
The effect of Cu treatments on growth in *Erica australis* plants (mean ± standard deviation, *n* = 4).

**Figure 2:**
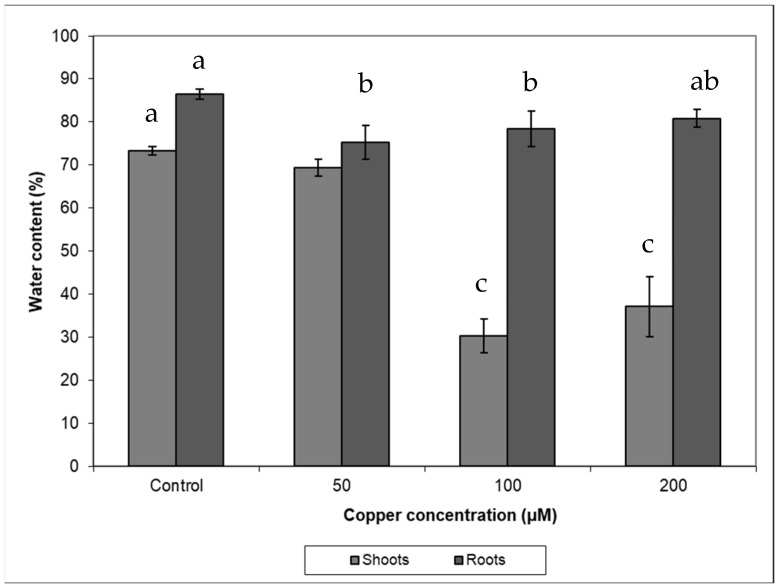
The water content in plants of *Erica australis* subjected to different Cu treatments (mean ± standard deviations, *n* = 4). Different letters indicate significant differences between groups (ANOVA, post hoc Tukey test, *p* < 0.05).

**Figure 3:**
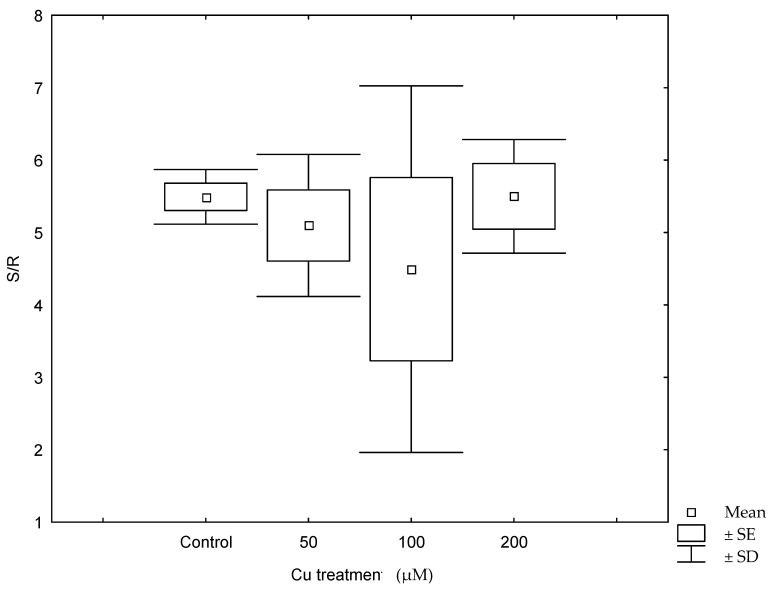
The shoot/root ratio (S/R) in plants of *Erica australis* after 30 days of growth in nutrient solutions with different Cu concentrations. SE, standard error.

Copper treatments negatively affected plant growth and biomass production (Figure 1, Table 1). The growth of control plants followed an exponential model (R^2^ = 0.979; y = 99 × e^0.023t^) while in those plants treated with 50 µM Cu, growth followed a linear relationship with time (R^2^ = 0.753; y = 99 + 1.24 × t). When plants were treated with 100 µM Cu, they stop growing after 10 days of treatment, while at 200 µM Cu, plants lost weight progressively, and biomass significantly decreased in both 100 and 200 µM Cu treatments. Darkened roots and wilted and bronzing leaves were observed in plants at the highest Cu concentration. A significant reduction in biomass was also observed when plants were treated with 50 µM Cu comparing with control (Table 1). A negative effect was observed between Cu treatments and shoot and root water contents (Figure 2), and the decrease in water content in all Cu treatments was statistically significant with respect to the control (*p* < 0.05). This decrease was more remarkable in shoots at the highest Cu concentration. The shoot/root ratio was not different among Cu treatments (Figure 3, *p* > 0.005). The malondialdehyde (MDA) content, as a marker of lipid peroxidation, was similar in roots from all treatments (Table 1), while in leaves, an increasing trend in MDA contents was observed, and a significant difference was found in MDA contents in leaves from plants cultivated at the highest Cu concentration. Catalase activity in the roots also significantly increased at 100 and 200 µM Cu concentrations. An increase in peroxidase (POD) activity in roots was found at 100 µM Cu, but POD activity decreased in plants cultivated at the highest Cu concentration (Table 1). Photosynthetic pigments were differently affected by Cu treatments (Table 1). The content of chlorophylls showed no differences among Cu treatments, whereas the content of carotenoids decreased only at the highest Cu concentration.

The Cu accumulation in the different organs (Table 2) followed the same pattern in control and treated plants (roots > stems ≥ leaves). Copper concentration in roots, stems and leaves (y) increased exponentially with Cu treatments (x) up to 100 µM Cu (y = a × e^bx^; Table 2). In the roots, Cu concentration also increased at the highest Cu treatment (*p* = 0.011), while in stems and leaves, the increase was not significant at 200 µM Cu concentration (*p* > 0.05). The concentration of macro and micronutrients in leaves, stems and roots is shown in Table 3. Copper treatments did not lead to mineral deficiencies in leaves, and a positive correlation was found between Cu treatment and almost all macro and micronutrients (Table 4). A similar trend was observed in roots except for K, where Cu showed an antagonist effect on root K content.

**Figure 4:**
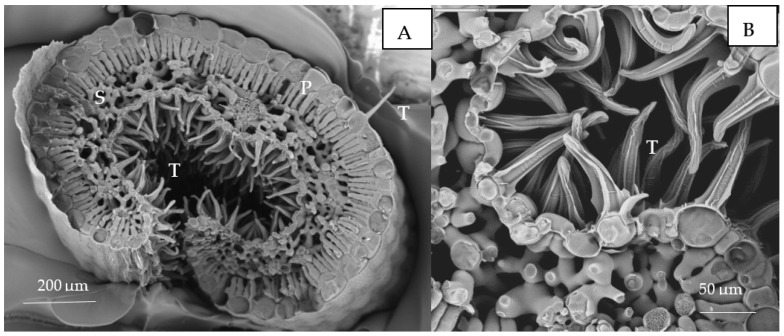
Leaves of *Erica australis* treated with 50 µM Cu by SEM observations. (**A**) Details of palisade (P) and spongy mesophyll (S) and adaxial trichomes (T); (**B**) Abaxial surface with trichomes (T).

**Figure 5:**
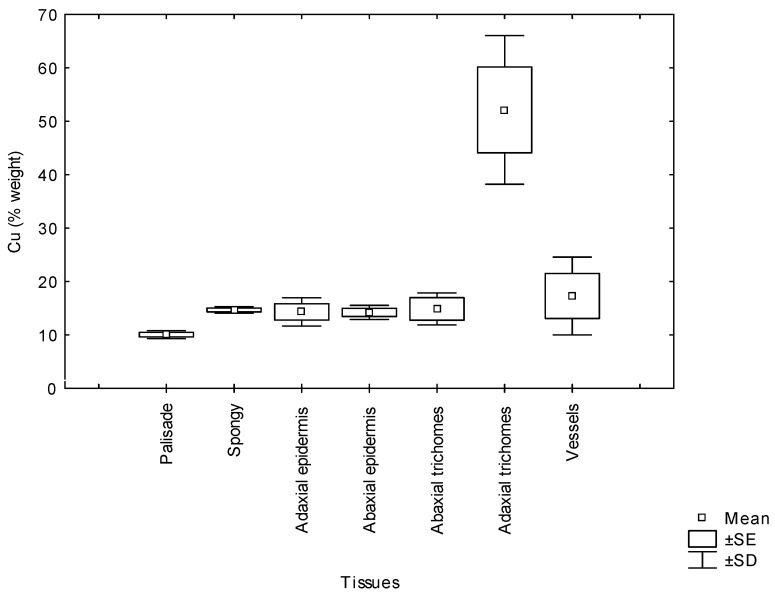
The copper concentration (% weight mean ± SD) in leaves tissues of *Erica australis* grown in culture solution added with 50 µM Cu. SE; standard error.

Morphologically, *Erica australis* presents small leaves with strongly revoluted margins (Figure 4A) and an epidermis covered by a thick cuticle and pluricellular and glandular trichomes (Figure 4B). The Cu content (weight %) in the different leaf tissues obtained by cryo-SEM/EDX is shown in Figure 5. The leaf adaxial trichomes (hair) accumulated significantly more Cu than other leaf tissues like parenchyma cells or epidermis. In the roots, Cu was homogeneously distributed in epidermal cells, cortex and vascular tissues (*p* > 0.05).

## 3. Discussion

*Erica australis* colonize the abandoned mining area of Riotinto, one of the most extensive examples of an extremely acidic environment [15] where soils are considerably contaminated by Cu [12]. In controlled conditions, plants survived at 200 µM Cu concentration in the medium but did not grow, and their leaves lost turgor as a consequence of decreased water uptake. At 250 µM Cu, the plants died after 20 days (personal observation). The root is the organ in direct contact with metal ions in the growth medium, and the accumulation pattern showed that Cu was almost immobilized in roots (Table 2). This is the most common mechanism of metal tolerance of metallophytes, which restricts metal transport into aerial parts [16]. In plants treated with the highest Cu concentration, the root Cu content was very high (5978 mg kg^−1^) compared with the Cu found in the root cortex of *E. australis* growing in the Riotinto mining area [12]. As presented in Table 2, similar values of root Cu concentrations have been reported by Monni et al. [4] in another species of Ericaceae under Cu treatments, but in contrast to our results, the plants showed a mortality rate of 60%.

The cryo-SEM/EDX analysis in the roots of Cu-treated plants revealed that Cu was uniformly localized among cortex, vascular tissues and rizodermis, but it is possible that Cu remained compartmentalized in the cell walls or vacuoles as was suggested as a strategy to cope with absorbed Cu [2,17,18]. Other mechanisms such as Cu accumulation in cytoplasmic vesicles are also observed [17]. High Cu concentration damage epidermal cells, reducing mitochondria and inducing cortical cell death [17]. There are gene families that play a key role in controlling Cu stress [19], and some of them are related to actin and cytoskeleton formation, metal transporters and superoxide dismutase activity in root tissues [20]. The root tissues of rice seedlings accumulate over 40% of the Cu present in the medium, and 60% of it was not fully available for transport [21].

Under field conditions, Monaci et al. [12] found that leaf Cu concentration in *E. australis* from Riotinto was similar to the values found in the control plants (grown on non-contaminated soils, 4.62 mg Cu kg^−1^), despite the high content of Cu in Riotinto soils (158 mg Cu kg^−1^). Their results showed the species was able to avoid Cu translocation from roots to leaves. Our results under controlled conditions demonstrate that the species was able to control upward transport of Cu as leaf metal concentration was only 3.5–6.5% of total Cu translocated to the shoots. In spite of this, Cu concentration in Cu-treated plants reached values higher than the normal concentration (10 mg kg^−1^) [22] or levels considered toxic (above 20–30 mg kg^−1^, 3). Some species can tolerate greater metal concentration in leaves, reaching up to 100 and 180 mg kg^−1^ Cu [23]. In the Riotinto mining area, the maximum leaf Cu concentration found in this species was 6.57 mg kg^−1^ [24]. Different environmental conditions and root colonization by mycorrhiza might play a role in metal tolerance. Even if the roots continued accumulating Cu at the highest Cu treatment, in the shoots, the Cu concentration did not increase further after 100 µM Cu (Table 2). A similar pattern was observed in *Avicennia marina*, where root structure alteration was not observed as a general symptom of Cu toxicity but darkened roots observed in plants cultivated at 200 µM Cu might indicate necrosis [2]. Root anatomical and physiological alterations play an important role in metal transport and plant growth [6,25]. The high Cu concentration in roots was responsible for the inhibition in plants growth and biomass production as a consequence of Cu toxicity. In fact, changes in biomass and in growth parameters have been indicators frequently used to test Cu toxicity [13,26,27]. Shoots showed similar sensitivity to roots to high Cu concentration in the nutrient solution (Figure 3). The reduction in biomass might be the result of damages produced by Cu stress in cell membranes by the increase in the H_2_O_2_ level that causing lipid peroxidation and further damages in organelles, nucleic acids, proteins and carbohydrates [13,28,29]. The shoot and root water content decreased significantly in plants treated with Cu (Figure 2). This was particularly remarkable in leaves from plants at the highest Cu concentration reflecting the metal interference in root water uptake and transport. The lower water contents in the aerial parts might be related to the reduced root growth and/or a consequence of changes induced in water transport proteins [30,31] and direct damages of root cell membranes [32].

Macro and micronutrients contents in shoots and roots tissues were not very much affected by Cu stress as it has been published for other species [2,4,14,33]. The exception was root K content, whose reduction might be related to membrane damage in root cells (see Table 3). The lack of antagonistic effects of Cu over other mineral nutrients might be another strategy of this species to cope with Cu toxicity.

On the other hand, in the leaves, the high Cu concentration did not affect photosynthetic pigment contents such as chlorophyll a and b (Table 1). Chlorosis was not observed in any of the Cu treatments in spite of being a frequent symptom of Cu toxicity in other plants [3,34]. In fact, nutrient elements, such as Fe and Mg, whose Cu-induced deficiency caused chlorosis [3], increased in leaves from Cu-treated plants. Certainly, the accumulation of Cu in mesophyll cells was low compared with trichomes in epidermal cells (Figure 5) which might avoid lipid peroxidation and membrane damage in chloroplasts. A similar tolerance strategy consisting of metal compartmentation to avoid metal toxicity has been found in *Erica andevalensis* [14] and in other species [35,36,37,38]. Carotenoids were negatively affected by Cu treatments, as reported by other authors [39,40], suggesting that these pigments were more sensitive to Cu-induced peroxidation. Peroxidase and catalase activities were used as stress markers since these enzymes may scavenge the high ROS production induced by high free metal cellular contents [13]. Once the metal is absorbed and accumulated in the cytosol, it may cause oxidative stress through the production of reactive oxygen species [6]. The free metal may be chelated with amino acids and then removed by compartmentation [2,27], avoiding further cell damages. Root peroxidase activity was increased at 100 µM Cu treatment, but it decreased at higher Cu concentration, making it difficult to conclude this enzyme might play a role in Cu tolerance. Some authors also proposed that an excess of Cu may decrease antioxidant capacity [1,41,42,43,44] Meanwhile, catalase activity certainly might be involved in the alleviation of oxidative stress caused by Cu (Table 1) in addition to other enzymes such as superoxide dismutases [45]. Malondialdehyde (MDA) is an indicator of oxidative damage in membrane lipids, and at the highest Cu concentration, an increase in MDA in leaves was found. Similar results were reported in other researches where the formation of free radicals by an excess of Cu react with membrane lipids to form lipid radicals and the cytotoxic MDA [40,46]. Present results suggest that even if *E. australis* may survive at a Cu excess of 200 µM by acting as a metal excluder species, its mechanism for survival breaks down (or is not enough) at high external Cu concentrations (higher than 50 µM Cu). At the threshold of 50 µM Cu, the tolerance capacity is disrupted, and Cu enters into the cytoplasm and generates oxidative stress.

## 4. Materials and Method

### 4.1. Plant Culture and Cu Treatments

Seeds of *Erica australis* proceeding from Tinto River (SW Spain) sterilized in 0.3% Na-hypochlorite and washed 3 times with sterile distilled water and pre-treated with heat (80 °C during 10 min) to promote germination. Then seeds were sown into tubes filled with rockwool and transferred into 10 l plastic containers with nutrient solution (pH 4.0) containing (in mM): NO_3_^−^, 5; H_2_PO_4_^−^, 1; SO_4_^2−^, 2.5; K^+^, 4; Ca^2+^, 2; Mg^2+^, 1. Micronutrients were supplied as prescribed in the Long Ashton nutrient formula [47], and Fe was provided as 4 mg/l Fe-EDDHA (ethylenediamine di-2-hydroxyphenyl acetate ferric). Plants were cultivated in a growth chamber with cycles of 26–23 °C (day–night temperature) and 16 h light/8 h darkness. When plants were three months old, they were treated with different Cu concentrations (50 μM, 100 μM and 200 μM) as CuSO4. The control treatment contained 1 μM Cu. These concentrations were selected after previous survival assays and according to soil Cu concentration found in the Riotinto mining area [12]. The nutrient solutions were continuously aerated with an aquarium air pump and renewed every 10 days to maintain a rather constant nutrient supply and metal concentration. The experiment was carried out for 30 days, and plants were weighed at a 10-day interval. All treatments had four replicates.

### 4.2. Growth Measurements and Elements Concentration

At the end of the experiment, plants were harvested, and plants were separated into roots, stems and leaves. Samples were washed once in tap water before being gently washed twice with distilled water and oven-dried at 70 °C for 48 h, and dry biomass was determined. Dried plant material was then milled and digested with a mixture of HNO_3_ and H_2_O_2_ [48]. Macro and micronutrients concentration in each plant part was determined by inductively coupled plasma atomic emission spectrometry (ICP-AES). The plant growth rate was assessed by fresh weight determinations every 10 days till the end of treatment (30 days). Water content (WC) in roots and shoots (leaves + stems) was calculated at harvest as WC = ((fresh weight − dry weight)/fresh weight) × 100. Plant shoot/root ratios (S/R) were calculated by dividing shoot fresh weight by its corresponding root fresh weight.

### 4.3. Determination of Biochemical Traits and Cu Localization by Scanning Electron Microscopy

Chlorophylls *a* and *b* and carotenoids were extracted from young shoots with 90.5% methanol and determined according to Lichtenthaler and Buschmann [49]. The activity of peroxidase (POD) was measured in aliquots of crude extracts from shoots and roots in a reaction mixture containing 0.1 mM H_2_O_2_ and 20.0 mM pyrogallol. The H_2_O_2_-dependent oxidation of the donor was followed at 430 nm (due to purpurogallin, ε_430nm_ = 2.47 mM^−1^ cm^−1^) as reported elsewhere [50]. The activity of catalase (CAT) was determined by measuring the disappearance of H_2_O_2_ following the decrease in absorbance at 240 nm following Aebi [51]. The reaction mixture contained 50 mM potassium phosphate buffer (pH 6.5), 1 mM EDTA and 15 mM H_2_O_2_. Protein concentration in the extracts was determined according to Bradford [52]. Lipid peroxidation was determined by estimating malondialdehyde (MDA), which was quantified according to Heath and Packer [53]. To localize Cu in different tissues plants, *E. australis* treated with 50 µM Cu was observed with Cryo Scanning Electron Microscopy (SEM), and energy dispersive X-ray (EDX) analysis was performed. Roots and leaves were cut into small pieces and frozen in nitrogen slush (−210 °C), quickly transferred to the cryospecimen chamber, freeze fractured and etched at −90 °C for the time required to remove surface ice. Samples were Au coated and examined using a Zeiss DSM 960 at −130 °C coupled with an energy dispersive X-ray microanalyzer. The microscopy was operated at an acceleration voltage of 25 kV with a beam current of 80 μA, a working distance of 12 mm, beam penetration depth of 5–6 μm and spectra collection time over 50 s. Analyses were carried out with a 10,000× magnification. All semi-quantitative values were normalized excluding C, Au and O [54] and were expressed as percent weight. All determinations were made for quadruplicates.

### 4.4. Statistical Analysis

Statistical analysis was performed with the Statsoft package v 6.12. The normality of the data was checked by Shapiro–Wilk test. A one-way analysis of variance (ANOVA) was used to detect significant differences between treatments, between Cu in plant tissues and parts, followed by Tukey’s HSD as a post hoc test (*p* < 0.05). Data were tested for normality, and in some cases, logarithmically transformed data were used in order to get a normal distribution. When data did not achieve homogeneity, the Kruskal–Wallis non-parametric test was used. A correlation analysis (Pearson) was performed between the Cu concentration in the nutrient solution and the other elements in the different plant parts. Regression analysis was used to determine several models (linear and exponential equations) of the plant concentration-response curves exposed to different Cu concentrations.

## 5. Conclusions

The exposure of *Erica australis* to 200 Cu µM did not affect plant survival, but a Cu concentration beyond 50 µM Cu caused a reduction in plant growth, indicating a Cu toxicity stress that caused an increase in catalase content in roots and lipid peroxidation (MDA) in leaves. The species accumulated Cu mostly in the roots (exclusion strategy), avoiding metal translocation to the aerial parts, but the Cu excess induces a decrease in K. Even though metal translocation was limited by root fixation, the species presented a high Cu concentration in the leaves (above the Cu toxicity threshold of many other species) but without a significant reduction in chlorophylls content or deficiency in macro and micronutrients. The accumulation of Cu in the abaxial trichomes of leaf epidermal cells might provide additional tolerance to metal toxicity.

## Figures and Tables

**Table 1:** Biomass, photosynthetic pigments and biochemical parameters measured in *Erica australis* treated with different Cu concentrations (mean ± standard deviation).

Cu (µM)	Biomass (g)		MDA (nmol/g Fw)	EA Root (U/mg Proteins)		Pigments (µg/g Fw)		
		Leaf	Root	CAT	POD	Chl.a	Chl.b	Carotenoids
**1 (Control)**	3.05 ± 0.85 a	295 ± 24.1 a	45.4 ± 31.4 a	<l.d.	3.20 ± 0.17 a	1548 ± 598 a	480 ± 232 a	317 ± 59.2 ab
**50**	1.15 ± 0.73 b	425 ± 284 a	24.5 ± 20.8 a	<l.d.	1.25 ± 0.23 b	1101 ± 38 a	341 ± 11.3 a	225 ± 6.20 bc
**100**	−0.17 ± 0.73 c	366 ± 51.4 a	5.59 ± 5.0 a	59.7 ± 16.6 a	3.91 ± 0.10 c	1623 ± 23 a	536 ± 13.8 a	400 ± 41.1 a
**200**	−0.32 ± 0.25 c	1668 ± 483 b	11.7 ± 0.32 a	91.6 ± 5.65 b	0.49 ± 0.14 d	1071 ± 362 a	694 ± 172 a	105 ± 70.2 c

MDA, malondialdehyde; EA root, enzymatic activities in roots; CAT, catalase; POD, peroxidase; Chl.a, chlorophyll a; Chl.b, chlorophyll b; l.d., detection limit; Fw, fresh weight. Different letters indicate statistical differences between treatments.

**Table 2:** The copper concentration (mean ± standard deviation; mg/kg) in *Erica australis* (*n* = 4) treated with Cu. Regression coefficients (a, b) ± standard deviation.

	Cu Treatments (µM)				Regression (1–100 µM)		
	1 (Control)	50	100	200	R^2^	a	b
**Leaf**	4.55 ± 1.41	24.9 ± 4.47	58.3 ± 17.8	50.5 ± 26.5	0.913	5.0 ± 0.8	0.026 ± 0.003
**Stem**	3.94 ± 0.30	27.8 ± 5.80	182 ± 98.1	170 ± 115	0.954	4.0 ± 0.7	0.037 ± 0.003
**Root**	22.5 ± 4.55	804 ± 196	3738 ± 288	5978 ± 1380	0.943	30 ± 8	0.052 ± 0.004

**Table 3:** The concentration of macro and micronutrients in leaves, stems and roots of *Erica australis* plants grown in nutrient solutions with different Cu treatments (*n* = 4). 1 µM = Control.

Cu Treatments (µM)	Organs		B (mg/kg)	Ca (%)	Fe (mg/kg)	K (%)	Mg (%)	Mn (mg/kg)	Na (mg/kg)	P (%)	S (%)	Zn (mg/kg)
1	Leaves	Mean	74.0	0.20	81.0	1.65	0.20	114.5	327.00	0.50	0.17	13.5
		Median	74.0	0.22	88.0	1.59	0.20	119.5	336.50	0.48	0.17	13.5
		St. Dev	14.8	0.06	21.0	0.25	0.03	18.6	49.27	0.21	0.02	0.6
	Stems	Mean	27.5	0.14	45.5	1.99	0.14	113.3	378.00	0.47	0.07	10.5
		Median	28.5	0.15	44.0	1.98	0.14	113.5	360.50	0.46	0.07	10.5
		St. Dev.	6.1	0.03	13.6	0.04	0.02	26.0	57.18	0.09	0.00	1.3
	Roots	Mean	16.7	0.17	2831.5	1.59	0.11	86.8	706.00	0.69	0.16	33.3
		Median	15.0	0.16	2828.5	1.56	0.11	83.5	670.00	0.68	0.17	34.0
		St. Dev.	2.9	0.05	316.1	0.14	0.02	13.9	148.84	0.09	0.02	7.7
50	Leaves	Mean	63.3	0.25	74.3	1.77	0.21	109.5	444.00	0.39	0.22	19.0
		Median	64.0	0.25	67.0	1.79	0.21	111.5	444.50	0.39	0.22	18.0
		St. Dev.	5.9	0.03	16.6	0.16	0.02	13.7	83.45	0.05	0.02	2.0
	Stems	Mean	21.3	0.19	24.3	1.62	0.12	87.8	434.00	0.44	0.09	10.8
		Median	21.0	0.17	24.0	1.66	0.12	86.5	417.50	0.43	0.09	11.0
		St. Dev.	5.5	0.04	3.5	0.21	0.02	18.8	47.66	0.07	0.01	0.5
	Roots	Mean	22.8	0.36	14291	1.14	0.13	89.0	950.75	1.05	0.21	47.7
		Median	23.5	0.26	13720	1.15	0.13	91.0	886.00	1.05	0.20	48.0
		St. Dev.	3.6	0.26	3189	0.13	0.01	7.2	344.99	0.20	0.02	0.6
100	Leaves	Mean	80.8	0.55	118.5	2.73	0.30	169.8	751.00	0.51	0.43	20.8
		Median	78.5	0.56	117.5	2.69	0.30	169.5	705.50	0.50	0.42	20.5
		St. Dev.	11.4	0.14	18.9	0.46	0.03	14.1	160.32	0.02	0.12	2.1
	Stems	Mean	21.0	0.23	40.7	2.06	0.11	85.5	750.25	0.46	0.19	18.8
		Median	21.0	0.20	37.0	2.00	0.12	87.5	718.50	0.45	0.19	18.0
		St. Dev.	7.5	0.07	7.2	0.39	0.02	10.7	219.64	0.07	0.03	7.9
	Roots	Mean	48.8	0.63	37239	0.73	0.21	157.5	1579.25	1.87	0.24	53.0
		Median	49.0	0.67	36855	0.69	0.21	155.5	1605.50	1.99	0.24	52.0
		St. Dev.	3.8	0.23	2403	0.11	0.07	49.3	242.39	0.44	0.03	5.3
200	Leaves	Mean	75.2	0.27	80.7	1.62	0.20	110.0	650.17	0.46	0.22	20.8
		Median	87.0	0.26	73.5	1.52	0.20	98.5	628.00	0.45	0.21	16.5
		St. Dev.	20.7	0.05	20.0	0.29	0.02	34.3	165.09	0.09	0.03	9.6
	Stems	Mean	40.7	0.16	124.2	1.60	0.09	81.0	852.40	0.48	0.14	19.7
		Median	43.0	0.15	124.0	1.62	0.09	60.5	864.00	0.49	0.15	20.0
		St. Dev.	7.2	0.02	15.2	0.40	0.04	52.1	91.30	0.06	0.05	6.4
	Roots	Mean	90.0	0.51	62856	0.92	0.17	191.0	2752.50	3.73	0.37	132.0
		Median	90.0	0.49	63189	0.89	0.14	167.0	2551.50	3.47	0.31	116.0
		St. Dev.	0.0	0.12	8651	0.31	0.07	71.1	489.14	0.81	0.12	48.5

**Table 4:** The Pearson correlation (*p* < 0.05) between the Cu concentration and the other elements in different plant parts of *Erica australis* (*n* ≥ 16).

	Macronutrients					Micronutrients			
	P	K	S	Mg	Ca	Fe	Mn	B	Zn
Leaves	-	0.607	0.751	0.643	0.696	0.484	0.641	-	0.550
Stems	-	-	0.848	-		-	-	-	0.605
Roots	0.960	−0.558	0.889	0.591	0.577	0.982	0.843	0.939	0.809

## Data Availability

The data that support the findings of this study are available from the corresponding author, upon reasonable request.
